# Hyper and hypo attention networks activations affect social development in children with autism spectrum disorder

**DOI:** 10.3389/fnhum.2022.902041

**Published:** 2022-08-11

**Authors:** Maya Sabag, Ronny Geva

**Affiliations:** ^1^Department of Psychology, Bar-Ilan University, Ramat Gan, Israel; ^2^The Developmental Neuropsychology Lab, The Leslie and Susan Gonda Multidisciplinary Brain Research Center, Bar-Ilan University, Ramat Gan, Israel

**Keywords:** autism spectrum disorder, attention, alerting, orienting, neurodevelopment

## Abstract

Children with autism spectrum disorder (ASD) experience a range of social and non-social attention deficits. To date, most studies assessed the neurological framework or discrete behavioral traits related to one attention network, leaving a gap in the understanding of the developmental cascade affecting the inter-relations among attention networks in ASD in a pervasive manner. We propose a theoretical framework that integrates the behavioral deficits and neurological manifestations through a cohesive developmental prism of attention networks’ activations while assessing their impact on social deficits in children with ASD. Insights arising from the model suggest hyper-and-hypoactivation of posterior attention networks leads to an altered prefrontal anterior attention network weight in ways that conjointly impact social performance in ASD. This perspective on how attention networks develop and interact in ASD may inform future research directions regarding ASD and attention development.

## Introduction

Autism spectrum disorder (ASD) is characterized by pervasive social-emotional deficits, including difficulties in forming, and maintaining relationships, understanding intentions, emotions, and affect, and adequately using verbal and non-verbal communication (American Psychiatric Association, 2013). Children and adults with ASD are also frequently diagnosed with comorbid attention-deficit/hyperactivity disorders (ADHD; Yerys et al., 2019). However, behavioral attention outcomes differ between co-morbid ADHD in ASD and ADHD non-ASD cohorts (Yerys et al., 2019). This suggests that attention alterations in ASD are mediated by a unique neural developmental cascade in their distributed attention networks.

Generally, three discrete attention networks have been proposed and tested functionally and neuro-anatomically: alerting, orienting, and executive attention (Posner and Petersen, 1990; Fan et al., 2002), with subunits devised for different aspects within each network (Petersen and Posner, 2012). The alerting network, designed to increase and maintain preparedness to respond to stimuli, is measured by comparing responses to cued and non-cued stimuli (Raz and Buhle, 2006), and is divided into phasic and tonic alerting (Krieber-Tomantschger et al., 2022). The phasic pathway, geared for increasing alertness, involves left hemispheric, thalamic, and brainstem regions (Sturm and Willmes, 2001). The tonic pathway is set for maintaining alertness by activation of the right hemispheric, thalamic, and brainstem regions (Petersen and Posner, 2012). The phasic and tonic alerting subunits typically display high inner-network connectivity during childhood, suggesting over-representation of bottom-up process’ affecting the development of the alerting network during infancy (Mullane et al., 2016).

The orienting network, representing the ability to select and follow the stimulus of interest (Raz and Buhle, 2006), involves dorsal and ventral pathways (Petersen and Posner, 2012). The dorsal pathway, in charge of bottom-up attention shifting (Majerus et al., 2018), activates parietal and frontal lobes, including the frontal eye fields (FEF) and the intraparietal sulcus (Farrant and Uddin, 2015; Farrant and Uddin, 2016). The ventral pathway enables top-down target following (Petersen and Posner, 2012), by activating the right ventricle and parietal regions, including the temporoparietal junction (TPJ) and the ventral frontal cortex (VFC; Fan et al., 2002; Farrant and Uddin, 2015).

The executive attention network includes two top-down regulatory pathways. The frontoparietal pathway regulates behavioral initiation and adjustment (Petersen and Posner, 2012). It is estimated by the difference in response to congruent and incongruent cues (Raz and Buhle, 2006). This pathway includes the dorsolateral prefrontal cortex and medial cingulate cortex. The cingulo-opercular pathway regulates maintenance of performance self-monitoring (Petersen and Posner, 2012). This pathway’s efficacy is linked to activation of mid-frontal and lateral prefrontal regions (Fan et al., 2002), specifically the anterior cingulate cortex (ACC) and the thalamus.

The neural and behavioral aspects of alerting, orienting, and executive attention have different developmental trajectories (Pozuelos et al., 2014). Alerting and orienting networks, the systems geared to perceiving external cues, comprise the posterior attention network (González et al., 2001). This network emerges early in infancy, reaching full maturation around 8 years (Colombo, 2001; Keehn et al., 2013; Krieber-Tomantschger et al., 2022).

The anterior attention network, comprised of the executive attention network (Davis et al., 2002; Krieber-Tomantschger et al., 2022), buds in early infancy (Colombo, 2001) when novelty seeking is already present but has a protracted developmental trajectory expressed in growing activation and connectivity as a function of age (Burstein et al., 2021). The anterior attention network presents significant development between 3 and 7 years, reaching full maturation only in adulthood (Rueda et al., 2005; Rothbart et al., 2011).

Geared to support goal-oriented behavior, the protracted development of the anterior attention network offers a growing moderating effect on alerting and orienting networks activation as a function of age (Jennings et al., 2007; Rothbart et al., 2011). This moderation effect is evident by enhanced prefrontal activity and reduced posterior activity as we grow older (Smith et al., 2011) and stronger prefrontal cortex connectivity to other neural regions as a function of neural maturation (Truelove-Hill et al., 2020). Considering the findings regarding inter-relations between the attention networks for goal-oriented behavior and learning in typical development, it is important to explore these inter-relations in populations that display atypical regulation of attention, maturation of neural pathways, and behavioral deficits such as ASD.

The literature on attention in ASD suggests an incoherent profile that may signal an altered developmental trajectory. Among the predominant attention features noted, ASD is characterized at young ages by ineffective attention orienting (Amso et al., 2014; Mutreja et al., 2016) and elevated alerting irrespective of stimuli importance (Liss et al., 2006; Keehn et al., 2013). These are accompanied by diminished executive attention moderation, with poor conflict resolution regarding which stimuli should receive priority and recruit more of our attention resources (Mutreja et al., 2016).

We suppose that ASD exhibits altered maturation trajectories of attention networks from childhood to adulthood (Murphy et al., 2014). Findings point to hyperconnectivity in childhood and hypoconnectivity in attention networks in adulthood (Farrant and Uddin, 2016). These findings suggest the importance of considering an evolving deficient inter-connectivity among the attention networks when children with ASD mature. Delineating behavioral manifestations corroborated by neural findings, here we delineate a model suggesting altered attention development cascade occurring in autism.

Recent literature describes ample neural findings during resting state (Farrant and Uddin, 2016; Delbruck et al., 2019; Jung et al., 2019), and connections between neural connectivity and autistic traits (Delbruck et al., 2019). We propose a theoretical framework designed to bind these deficits together. We postulate that children with ASD present an idiosyncratic development of the attention networks whereby early on in life, they confront posterior attention network hyperconnectivity (Minshew and Keller, 2010), posing difficulty in refining behaviors to important stimuli due to overwhelming activation of bottom-up attention networks. Consequently, as neural networks of children with ASD develop, early hyperconnectivity turns to hypoconnectivity (Farrant and Uddin, 2016). These deficits are moderated by dysregulated development of the executive attention pathways (May and Kana, 2020) that limit flexible exploration and learning. Considering this theoretical supposition, we examine the behavioral and neural literature, piecing together both aspects into a cohesive model.

## Evidence concerning the arousal attention network: Behavioral

Taking into account the maturational changes expected at the various levels of the neural system, we were recently able to put forth the brainstem informed autism framework, highlighting that arousal deficits described in ASD are due to poor bottom-up regulation (Burstein and Geva, 2021). The behavioral outcomes of alerting attention in children with ASD may seem inconsistent. While some studies report alerting behaviors in children and adolescents with ASD to be intact (Keehn et al., 2010; Fitzgerald et al., 2015), others demonstrated elevated arousal (Hames et al., 2016). These differences may be related to age differences across samples. Elevated arousal in ASD is evident by wider pupil dilation in response to alerting salient cues (Geva et al., 2013), overall hyperarousal, and deficits disengaging from salient stimuli seen in young infants at-risk for ASD (Elsabbagh et al., 2013; Zivan et al., 2021). These findings suggest hyperreactivity to peripheral stimuli and unregulated bottom-up processes that compromise the efficacy of children with ASD in reacting adaptively to environmental demands early in development. Fitting with this notion, alerting performance has been linked to social impairments in ASD (Fitzgerald et al., 2015), emphasizing the impact of alerting attention development on the core deficits of ASD, possibly implying specific neural correlates of the alerting attention network to their behavioral responses.

## Evidence concerning the arousal attention network: Neural

Elevated physiological hyperarousal (Geva et al., 2013) is expected to involve the brainstem, thalamus, and the left cortical mantel, representing hyperactivation of the phasic alerting pathway. Indeed, children with ASD demonstrate stronger connectivity in limbic and somatosensory regions relative to typically developing children (Jung et al., 2019). They also present with diminished fusiform cell count in the brainstem (Kulesza et al., 2011), which corroborates deficits in arousal of this population (Geva et al., 2017). Integrating these data suggests that deficits in brainstem activity are linked to alterations in alerting of attention, gaze engagement, and social attention seen in infants at higher risk for ASD (Smith et al., 2019). For example, the diminished volume of fusiform cells in the medial-superior olive and superior paraolivary nucleus (Kulesza et al., 2011) and pulvinar activation (Kleinhans et al., 2011) may influence delayed arousal to social cues and diminished orienting to such stimuli at infancy.

The specific effects of these alterations that unfold in time are yet unresolved. Findings from ASD cohorts indicate diminished neural learning and synaptic pruning earlier in development (Belmonte et al., 2004) and unexpected patterns of connectivity between different neural networks (Fitzgerald et al., 2015)- suggest cascading effects of the phasic alerting network on the orienting and executive attention networks.

## Evidence concerning the orienting attention network: Behavioral

Findings regarding orienting responses in ASD are inconsistent. Some note deficient or diminished orienting (Keehn et al., 2010; Hames et al., 2016; Mutreja et al., 2016), while other studies suggest intact, or augmented behavioral orienting to both social and non-social cues (Greene et al., 2011; Fitzgerald et al., 2015). Plausibly, the orienting network is a less distinctive characterizing feature of childhood ASD. Yet reports of deficient social orienting in ASD being correlated with symptom severity (Latrèche et al., 2021) call for a deeper dive into the neural findings concerning the orienting network activation.

## Evidence concerning the orienting attention network: Neural

**FIGURE 1:**
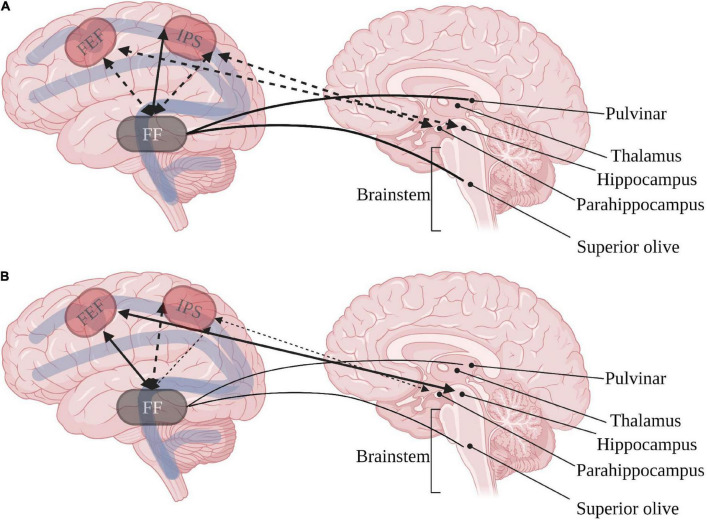
Inter-relations between dorsal orienting (red) and arousal networks in **(A)** typical development and **(B)** ASD. Continuous arrows represent positive correlations, dashed arrows represent negative correlations. FEF, frontal eye field; IPS, intraparietal sulcus; FF, fusiform gyrus. Created with BioRender.com.

As expected from the inconsistent behavioral orienting of attention, connectivity of the orienting network to other brain regions may seem inconsistent. Nevertheless, some data point to a greater susceptibility of the dorsal orienting pathway as compared with the ventral pathway. ASD studies show that the FEF is positively, rather than negatively, connected to the left hippocampus and fusiform gyrus. In contrast, the ventral and posterior intraparietal sulcus’ present diminished negative connectivity to the left para-hippocampus and fusiform gyrus (Fitzgerald et al., 2015). The connectivity to the fusiform gyrus has special importance due to evidence of diminished fusiform cells in the superior olive (Kulesza et al., 2011) and the relation of fusiform and pulvinar activation to social gaze aversion in ASD (Kleinhans et al., 2011). These findings point to heightened co-dependence of the bottom-up dorsal orienting pathway and the phasic arousal pathway in ASD (see Figure 1).

**FIGURE 2:**
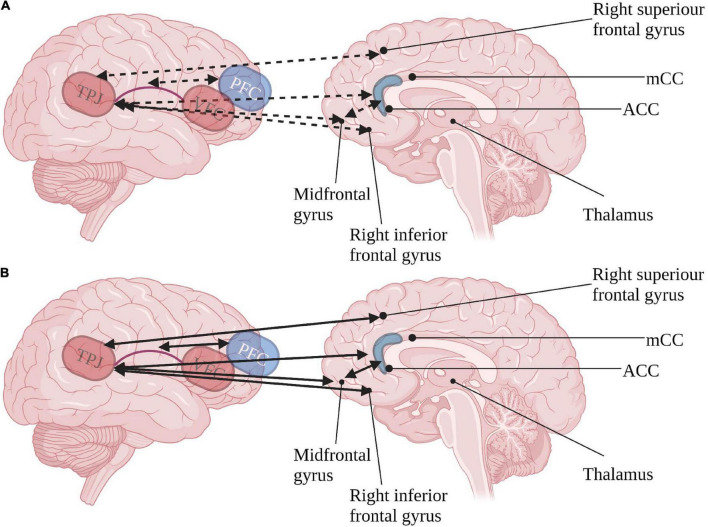
Inter-relations of ventral orienting (red) pathway and executive attention (blue) pathways in **(A)** typical development and **(B)** ASD. Continuous arrows represent positive correlations, dashed arrows represent negative correlations. TPJ, tempotoparietal junction; VFC, ventral frontal cortex; PFC, prefrontal cortex; ACC, anterior cingulate cortex; mCC, medial anterior cingulate. Created with BioRender.com.

The ventral pathway, concerned with top-down target following, seems to be highly affected in ASD. Inner-network hyperconnectivity during resting state, specifically in the right TPJ and VFC, has been documented (Chien et al., 2015; Farrant and Uddin, 2016). This pathway also produces co-activation with frontal regions associated with the executive pathways (Fitzgerald et al., 2015; Hames et al., 2016), and could account for hyperactivation in frontal regions of the mirror neuron system (MNS) consistently demonstrated in ASD (Chan and Han, 2020), resulting in altered self-monitoring (Fitzgerald et al., 2015) as described in Figure 2. Moreover, the TPJ, which is of special interest due to its involvement in social brain functions (Blakemore, 2008; Burnett et al., 2011), is related to social affect deficits in ASD (Delbruck et al., 2019). The unique hyperconnectivity of the TPJ with the MNS possibly hinders social learning and adaptiveness in ASD due to inadequate imitation (Williams, 2008; Fitzgerald et al., 2015). This presumably outlines an ineffective social orienting feedback loop, resulting in a cascade of diminished social internalizing of social engrams, furthered by a decrease in social interest and social orienting.

Integrating the involvement of the dorsal pathway affecting phasic arousal and the augmented connectivity between the ventral pathway and executive pathways may indicate patterns of pervasive dependence instead of titrated dependence between the three attention networks in ASD. This highlights the importance of considering the links between the posterior alerting and orienting networks to the anterior executive attention network in ASD.

## Evidence concerning the executive attention network: Behavioral

Executive functions play an important role in decision-making, adaptive behavior, and self-regulation. Thus, altered development and activation of the executive attention network has an important impact on refining alerting and orienting of attention (Rothbart et al., 2011) along with social competence of children with ASD (May and Kana, 2020). Given the postulated inter-relations between the attention networks, it is not surprising that behavioral findings concerning the executive attention networks in children and adolescents with ASD are also inconsistent. While some report typical executive attention (Keehn et al., 2010; Hames et al., 2016), others note behavioral deficits (Mutreja et al., 2016). These heterogeneous characteristics of participants with ASD are expected to correlate with neuronal activations.

## Evidence concerning the executive attention network: Neural

Parent reports of executive attention expressions have been found to correlate with executive attention network activation only in ASD (Samyn et al., 2014), demonstrating the unique importance of altered neural activation on their behavioral outcomes. Considering the prefrontal cortex development, which promotes executive processes, ASD presents in childhood with enlarged frontal lobes (Carper and Courchesne, 2000; Carper and Courchesne, 2005), supporting the notion that insufficient synaptic pruning early-on-in-life impacts the executive attention network’s efficiency. Lowered executive attention network efficiency in ASD is evidently the product of hyperactivation in the ACC (May and Kana, 2020) and positive rather than negative connectivity of the ACC and precuneus in ASD (Fitzgerald et al., 2015). Correspondingly, executive attention produces co-activation instead of a moderated activation of the ventral orienting pathway in children with ASD (Fitzgerald et al., 2015). This deficient interaction among the attention networks thus plays a primary role in the low conflict resolution as to where and to what extent one should pay attention to a cue, leading to altered sensitivity to peripheral cues and decreased learning specificity in ASD.

## Alterations in inter-relation among attention networks in autism spectrum disorder

The alerting, orienting, and executive networks represent relatively separate constructs in neurotypical cohorts (Fan et al., 2002; Petersen and Posner, 2012) and activate independent neural pathways which interact and moderate one another (Fernandez-Duque and Posner, 2001). Functional imaging while performing attention tasks suggests neural pathways in ASD are less independent (Fitzgerald et al., 2015).

We suggest that contrary to neurotypical brain activity, the atypical development in ASD results in altered inter-dependence of the attention networks (see Figures 1, 2) due to synaptic pruning differences that accompany the reported gray and white matter changes. Phasic arousal seems to hinder effective orienting by the dorsal pathway (Fitzgerald et al., 2015). Synaptic pruning seen in typical development does not moderate the activity of the ventral orienting pathway in ASD adequately, and elevated activation of executive network regions coincides with deficient orienting behavior (Hames et al., 2016). Thus, it appears that children with ASD are characterized by hyperactivation of the posterior attention network (Farrant and Uddin, 2016), resulting in insufficient differentiation between central and peripheral cues and diminished neural susceptibility to top-down executive inputs.

In adulthood, the orienting pathways are characterized by hypoactivation, failing to maintain the cognitive overload, and developing inefficient activation patterns to external and internal cues (Farrant and Uddin, 2016). These are plausibly the consequence of diminished specialization of neural networks to their independent roles seen in typical cohorts. This developmental cascade results in ineffective learning of communication and social skills due to protracted activation of the posterior attention network that results in hypo/hyper activations of the executive attention pathways (May and Kana, 2020).

## Discussion

The proposed framework of attention network activation aims to examine how deviances in attention networks impact learning in ASD. ASD’s most prominent deficit, processing and responding to social cues (American Psychiatric Association, 2013), is considered a result of deficient social learning stemming at infancy. At this early stage of life, ASD presents with hyperactivation of the alerting network that compromises social functioning (Minshew and Keller, 2010; Geva et al., 2017), that elicits deficits in orienting to social cues (Hedger et al., 2020), and produces poor executive resolution of conflicting social information (Blijd-Hoogewys et al., 2014). The framework of attention network activation thus suggests an altered and pervasive inter-dependency among the attention networks in ASD that progress over time.

Findings in ASD show multiple positive inter-network correlations (Fitzgerald et al., 2015) that result in low specificity in network activation due to insufficient synaptic pruning during childhood. The data suggest that this hyperactivation of the attention networks early-on-in-life is predominantly of the posterior attention network, in the arousal and the ventral orienting networks (Farrant and Uddin, 2016; Geva et al., 2017). This hyperactivation produces low discrimination between high and low stimuli importance due to deficiency in executive network moderating effects (Rothbart et al., 2011). As a result, social schemes are not internalized efficiently, possibly due to altered connectivity of the TPJ with the MNS (Fitzgerald et al., 2015; Chan and Han, 2020), in ways that later in development, elicit hypoactivation of the attention networks (Farrant and Uddin, 2016).

Implications of this framework suggest that supporting more efficient social processing during early life in children at risk for ASD may mitigate over-activation of the alerting network by limiting overload and a sense of being overwhelmed. Support can be offered in different ways, aiming to highlight social processing while lowering peripheral stimulation, relieving the load on the orienting network; and/or lowering social salience to down-regulate the load on the alerting network. As such, intervention programs should focus not only on what but also on the context in which social targets are presented. Implications may inform an adjustable design of clinic rooms, classrooms, and even home environments to suit the here-and-now attentional capacities and social interaction goals.

The framework of attention network activation in ASD is a novel approach that needs further investigation in naturalistic settings. Most data analyzed was obtained in laboratory settings, in resting state, or individualistic task-preforming settings. Future research should assess the proposed model longitudinally and explore the activation and connectivity of the attention networks in response to real-life social interactions. Such research will enable better understanding of the role of attention networks’ developmental trajectories on social deficits in ASD in ecological settings. In doing so, the cause of ASD’s social difficulties will be better understood and could be reduced with adequate scaffolding. Research in this field will pave the path for developing intervention programs that could diminish mental effort and ultimately improve quality of life in ASD.

## Data availability statement

The original contributions presented in this study are included in the article/supplementary material, further inquiries can be directed to the corresponding author.

## Author contributions

Both authors wrote the manuscript together and approved the submitted version.
